# Hair Growth Promotion Activity and Its Mechanism of *Polygonum multiflorum*

**DOI:** 10.1155/2015/517901

**Published:** 2015-07-30

**Authors:** Yunfei Li, Mingnuan Han, Pei Lin, Yanran He, Jie Yu, Ronghua Zhao

**Affiliations:** Yunnan University of Traditional Chinese Medicine, Kunming, Yunnan 650500, China

## Abstract

*Polygonum multiflorum* Radix (PMR) has long history in hair growth promotion and hair coloring in clinical applications. However, several crucial problems in its clinic usage and mechanisms are still unsolved or lack scientific evidences. In this research, C57BL/6J mice were used to investigate hair growth promotion activity and possible mechanism of PMR and* Polygonum multiflorum* Radix Preparata (PMRP). Hair growth promotion activities were investigated by hair length, hair covered skin ratio, the number of follicles, and hair color. Regulation effects of several cytokines involved in the hair growth procedure were tested, such as fibroblast growth factor (FGF-7), Sonic Hedgehog (SHH), *β*-catenin, insulin-like growth factor-1 (IGF-1), and hepatocyte growth factor (HGF). Oral PMR groups had higher hair covered skin ratio (100 ± 0.00%) than oral PMRP groups (48%~88%). However, topical usage of PMRP had about 90% hair covered skin ratio. Both oral administration of PMR and topically given PMRP showed hair growth promotion activities. PMR was considered to be more suitable for oral administration, while PMRP showed greater effects in external use. The hair growth promotion effect of oral PMR was most probably mediated by the expression of FGF-7, while topical PMRP promoted hair growth by the stimulation of SHH expression.

## 1. Introduction

Black and shiny hair is well recognized standard of human health in Asian countries. Hair loss and gray hair are considered as the early signs of aging or other chronic diseases. Although alopecia is common, neither life-threatening nor paining, it is a distressing disorder, even leading to a series of mental disease.

At present, clinical treatments of hair loss and other related problems are still needed to be solved. Many hair growth and hair dying accelerators in the market showed negligible effects. Finasteride and minoxidil are usually first line therapy for its treatment. However, finasteride may lead to impotence, loss of interest in sex, trouble having an orgasm, and abnormal ejaculation [1]. Topical usage of minoxidil may induce burning, stinging, or redness at the application site [2]. Moreover, high prices and unsustainable effects of finasteride and minoxidil also limit their widespread usage. Other options include topical or systemic usage of spironolactone or flutamide, although they have a high incidence of feminizing side effects and are better tolerated in female androgenic hair loss. These deficiencies force these hair losers and gray-haired ones to wear a wig or use hair dyes regularly. Hair dyes may be another choice; however, most of the artificial hair dyes contain harmful chemical ingredients, which could induce many serious skin problems. Therefore, searching for effective and safe hair growth promotion and hair dying drugs in relative low price from traditional Chinese medicine or natural products has enormous social and economic benefits.

*Polygonum multiflorum* Radix (PMR) and* Polygonum multiflorum* Radix Preparata (PMRP), traditional Chinese medicine originated from* Polygonum multiflorum*, had long history for hair growth and hair dying in clinical applications. Dating back to AD 812 years ago,* P. multiflorum* was recorded as an extremely tonic medicine that could “benefit for the essence, strong your spirits, protect your beauty, black your hair, and extend your life” (*Polygonum multiflorum *Biography, AD 812). In the* Compendium of Materia Medica* (Shizhen Li, AD 1578), the words “it could benefit the essence, kidney, spleen, bones and hair as a tonic traditional Chinese medicine” were used to describe it. However, little mechanisms were clarified. Only a few studies have shown that* P. multiflorum* extract could promote the quiescent C57BL/6J mice hair follicles to enter anagen stage earlier [3]. Topical usage of PMR increased experimental animals' hair follicle quantities and extended follicle length significantly. Sonic Hedgehog (SHH) protein and *β*-catenin expression levels were significantly increased in mice after topical application of PMR and fermented products. In another* in vitro* pig hair follicle cell culture model, PMR decoction could inhibit hair follicle apoptosis and retard the anagen stage from entering the catagen stage [4]. However, several crucial problems in the clinic usage and mechanisms of* P. multiflorum* are still unsolved or lacked of scientific evidence. Whether PMR or PMRP had better curative effect for baldness and canities was lacking research data. Moreover, the hair growth and hair dying mechanisms of* P. multiflorum* lacked relevant research.

Therefore, C57BL/6J mice were used in this research to investigate PMR and PMRP hair growth promotion activity and possible mechanism. Their extractions were administered orally and/or topically. Hair growth promotion activities were investigated by hair length, follicles numbers, hair covered skin ratio, and hair color. Several cytokines involved in the hair growth procedure were tested in our research, such as fibroblast growth factor 7 (FGF-7), SHH, *β*-catenin, insulin-like growth factor-7 (IGF-7), and hepatocyte growth factor (HGF).

## 2. Materials and Methods

### 2.1. Plants Materials and Chemicals

*P. multiflorum* Thunb. was collected in June of 2008 by the authors in Luquan county in Yunnan province and identified by Professor Ronghua Zhao of Yunnan University of Traditional Chinese Medicine. Voucher specimens were deposited in the Herbarium of Pharmacognosy, Yunnan University of Traditional Chinese Medicine.

### 2.2. Processing and Extraction of PMR and PMRP

**Figure 1:**
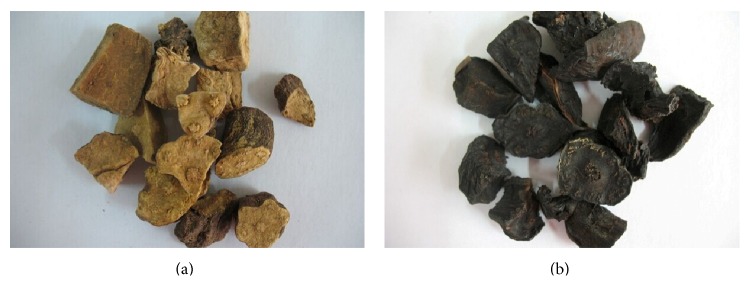
Photographs of PMR (a) and PMRP (b).

PMRP was steamed by the authors from PMR with black soybean decoction according to the procedure recorded in Pharmacopoeia of the People's Republic of China, 2010 edition [5] (Figure 1).

1000 g of PMR and 900 g of PMRP were decocted with water (10 times, 8 times, and then 6 times in volume) for three times, respectively. Extracts were combined, condensed, and lyophilized. The equivalent low, middle, and high dosages for mice of PMR and PMRP were of 0.2925, 0.5850, and 1.1700 g/kg body weight according to Pharmacopoeia of the People's Republic of China, 2010 edition [5].

### 2.3. HPLC-DAD Analysis of PMR and PMRP

All experiments were performed by using Dionex Ultimate 3000 HPLC system (Dionex Technologies, USA), which included a binary pump, an autosampler, a column oven, and a diode array detector plus on-line degasser. Data were analyzed with Chromeleon 6.8.

The separations were achieved on Zorbax SB-C_18_ analytical column (4.6 mm × 250 mm, I.D., 5 *μ*m particle diameter, supplied by Agilent Technologies, USA).

Gradient elution with mobile phase consisting of (A) 0.1% H_3_PO_4_ and (B) methanol was used. The nonlinear gradient elution program was utilized. Methanol percentage was 40% (in the initial time), 70% (5 min), 80% (10 min), 85% (15 min), 90% (20–25 min), and 40% (35 min). Detection wavelength was set at 254 nm. The oven temperature was set at 30°C and the flow rate was set at 1.0 mL·min^−1^.

References of 2,3,5,4′-tetrahydroxystilbene-2-*O*-*β*-D-glucoside (TSG), emodin, and physcion were weighed precisely and dissolved in methanol. Extracts of PMR, PMRP, and their total anthraquinone were weighted accurately and resolved in 10 mL 50%, 50%, and 100% methanol, respectively. A 10 *μ*L injection value was used in all the analysis. The peaks of TSG, emodin, and physcion were identified by comparing their retention time values and UV spectra with those of the standards.

### 2.4. Animals and Treatments

88 C57BL/6J male mice were provided by Beijing HFK Bioscience Co. Ltd. They were aged 6 weeks and weighed 20 ± 2 g. They were housed six to a stainless steel cage containing sterile paddy husk as bedding in ventilated animal rooms. They were acclimated in the controlled environment (temperature 22 ± 1°C; 60 ± 10% humidity; and a 12 h/12 h light/dark cycle) with free access to water and a commercial laboratory complete food. All animal experiments were performed in compliance with the animal experimental ethics committee of Yunnan University of Traditional Chinese Medicine (R-062012001). All reasonable efforts were made to minimize the animals' suffering.

**Figure 2:**
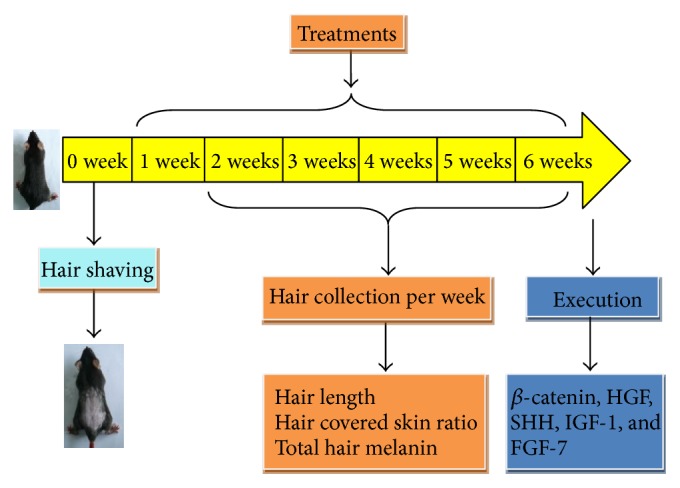
Schedule design for this research.

The mice were randomly assigned to 11 groups (*n* = 8) (Table 1) after adaptive feeding for three days. All animals were shaved (about 10 cm^2^ in the back) using animal clipper. Group A was the normal group with physiological saline orally administrated. Groups B, C, and D received low, middle, and high dosage of PMR orally. Groups E, F, and G received low, middle, and high dosage of PMRP orally. Groups H and I received topically usage of PMR and PMRP. Groups J and K received both oral and external usage of PMR and PMRP simultaneously. The whole schedule design for this research was shown in Figure 2.

### 2.5. Investigation of Hair Growth Promotion Activities

#### 2.5.1. Animal Hair Collection and Hair Length Determination

Collections of animal hair were performed from 2nd week to 6th week. Ten hairs were collected randomly from the shaving area in mice back and measured with Vernier caliper carefully.

#### 2.5.2. Number of Follicles

5 *μ*m sections of mice back skin were stained with hematoxylin and eosin (H&E). Digital photomicrographs were taken from representative areas at a fixed magnification of 100x. The H&E dyes were photographed using a digital photomicrograph and all of the images were cropped in a fixed area of 100 pixels width. Average hair follicles numbers were counted from representative areas.

#### 2.5.3. Determination of Hair Melanin

Hair samples of all mice in the same group were collected. 1 mL of water and 9 mL of Soluene-350 (a kind of strong organic solvent that could dissolve a variety of organizations, such as hairs) were added to 10 mg of hair sample [6]. The mixtures were heated twice at boiling temperature, for 30 and 15 mins, with a short cooling intermittence. Then they were centrifuged for 10 mins at 10000 rpm. The absorbance of the supernatant at 500 nm was measured by UV-Vis spectrophotometer (UV-4802H, Unico Instrument Co. Ltd., Shanghai).

### 2.6. Assessment of Related Proteins and Growth Factors in Skin

Mice skins were collected in the 3rd, 4th, and 6th weeks. Skin samples were disinfected with 75% ethanol and deposited immediately in −80°C for 24 h and then transferred to liquid nitrogen for storage.

Skin samples were weighed and washed with 0.9% saline. 100 mg of skin samples in each group was cut into pieces and then homogenated with 0.9 mL physiological saline. The homogenates were centrifuged for 10 min in 4°C. FGF-7, IGF-1, HGF, *β*-catenin, and SHH contents in the supernatant were measured by Elisa kits (CUSABIO Biotech Co. Ltd.).

### 2.7. Statistical Analysis

All data in this research were expressed in the form of mean ± SD. One-way analysis of variance (ANOVA) was performed when multiple group comparisons were carried out. The relationships between variables were assessed with Pearson's correlation coefficient.

## 3. Results and Discussions 

### 3.1. TSG, Emodin, and Physcion Contents in Crude Drug

**Figure 3:**
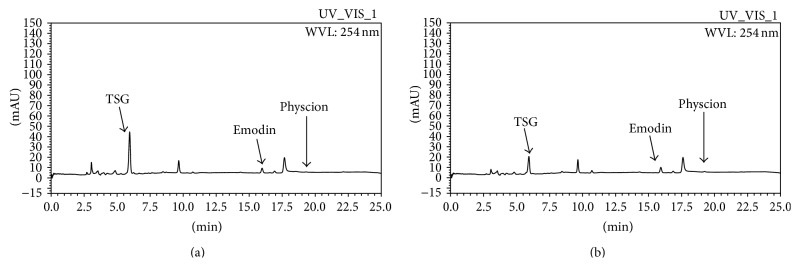
HLPC profiles of PMR (a) and PMRP (b).

Processing procedure induced chemical constituents changes were obviously revealed in the chromatography profiles before and after processing (Figure 3). TSG concentration reduced from 47.27 mg/g in PMR to 29.26 mg/g in PMRP. Tangible TSG reduction was observed after processing. 38.10% of TSG was reduced after processing procedure in water extraction. 343.76% emodin and 178.02% physcion were increased in PMRP compared to PMR.

### 3.2. Hair Length, Hair Covered Skin Ratio, and Hair Color

**Figure 4:**
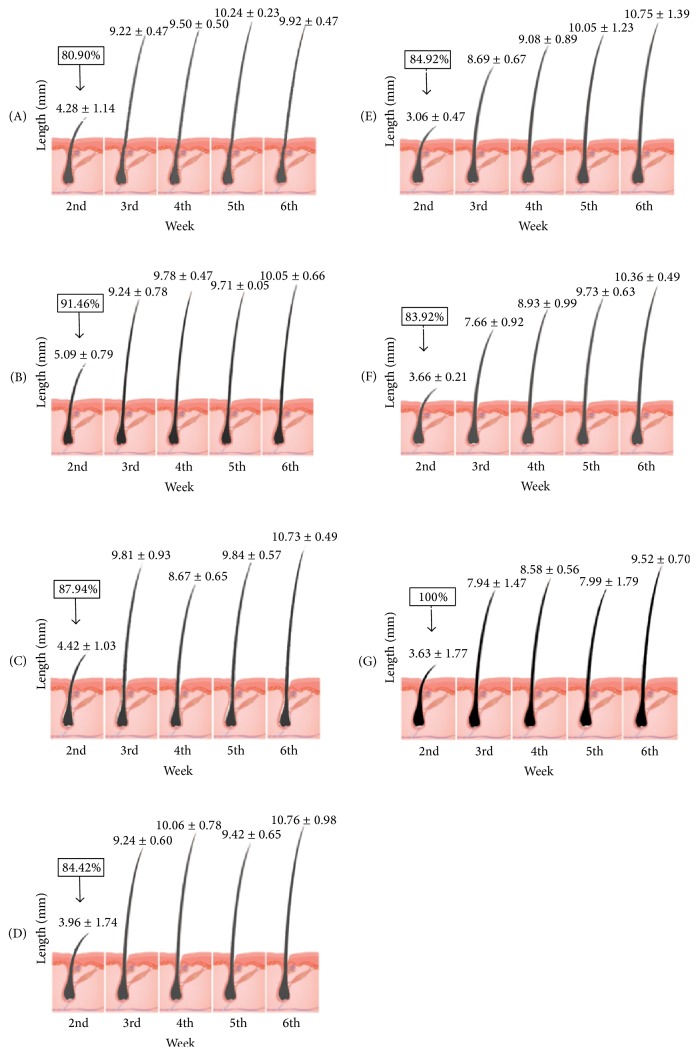
Hair lengths and melanin contents in oral groups. Note: *X* ± SD, mm, *n* = 10/per mice, *n* ≥ 3. Hair melanin contents, normalized with Group H as 100%, were listed in the black box located in the top left corner of each figure.

According to the hair length data (Table 2 and Figure 4), most of the hair growth was accomplished between the 2nd to 3rd week in all groups. Mice in different groups got similar hair length in the end of the research.

**Figure 5:**
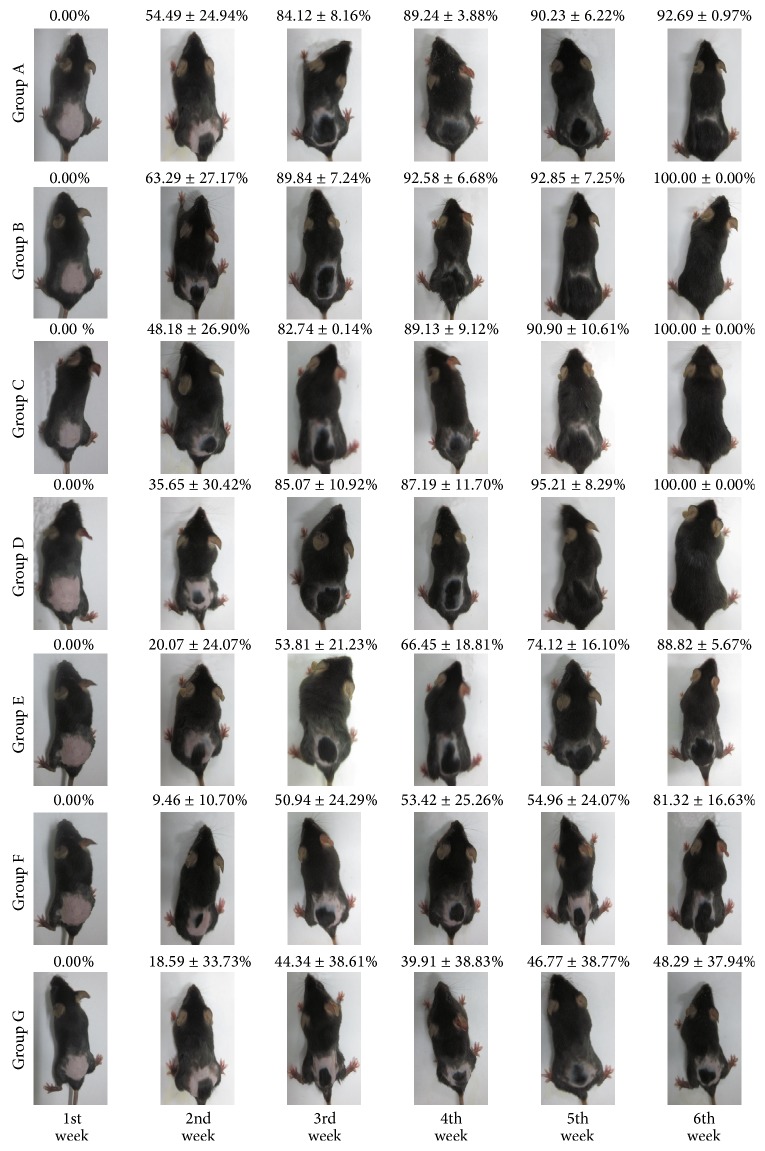
Hair covered skin ratio (%) from 1st to 6th week in oral groups. Note: *X* ± SD, mm, *n* = 10/per mice, *n* ≥ 3.

From the area of the hair covered skin observed (Figure 5), oral PMR groups (B, C, and D) showed better hair growth promotion effects than oral PMRP (E, F, and G) groups from 2nd to 6th week. PMR mice own 96%~98% hair covered skin ratio at the end of the research, while PMRP mice had about 46%~78% hair covered skin ratio, which were even less than the normal group.

**Figure 6:**
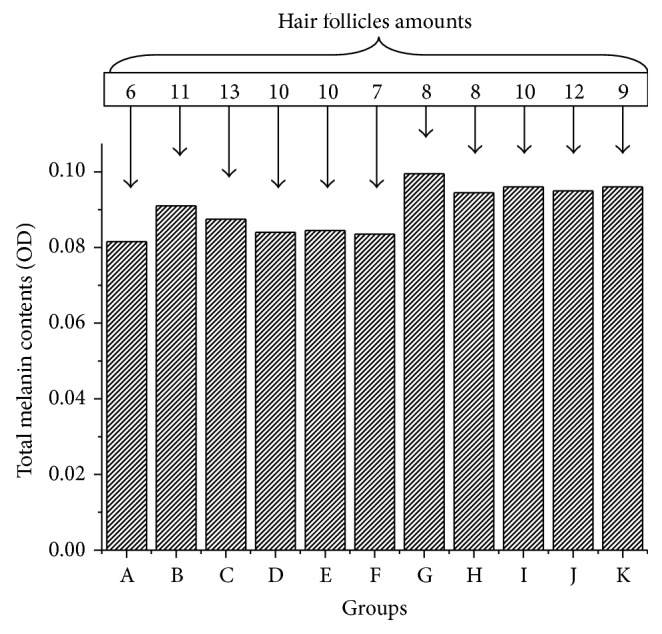
Hair melanin contents and hair follicles amounts in all groups.

Nevertheless, as can be seen from the determination of total melanin data (Figure 6), Group H (high dosage of PMRP) possessed the deepest color of hair. Comprehensively, judging from both the hair growth speed and area of hair growth, we could get a conclusion that oral PMP show great hair growth promotion activity.

### 3.3. Medication Time for Hair Growth

Mice hair in all groups showed the fastest growth speed from the 2nd to 3rd week. Hair length growth was almost accomplished at the end of the 3rd week; however, about six weeks were needed for 90% of the shaved skin to be covered by hair. Therefore, we recommended continuously taking of PMR for at least 6 weeks for the treatment of alopecia.

### 3.4. Relationship between Drug Delivery Route and Hair Growth Activity

For oral and topical drug delivery, no difference could be found in terms of hair length (Table 3). The hair color was a little darker in the topical delivery groups than the oral groups. Judging from the hair growth ratio, oral administration of PMR (Group C) showed better effect than topical administration of PMR (Group H). Nevertheless, PMRP was more suitable in topical delivery group (Group I) than oral delivery group (Group F). The reasons for this phenomenon were probably due to the main chemical compositions of* P. multiflorum* which were changes before and after processing (see Section 3.1).

From what has been discussed above, we found that oral and topical delivery routes were both possible effective ways for hair growth promotion drugs without significant efficiency difference. Regional concentrations of drugs in the hair follicles may be of similar values whether oral or topical routes were used.

However, the two delivery routes may be suitable for different drugs. In our results, PMR was considered to be more suitable orally, while PMRP showed greater effects in external use.

### 3.5. Dose-Effect Relationship of PMP and PMRP in Hair Growth and Hair Color

No dose-effect relationships of PMR and PMRP in hair length and hair covered ratio were observed. Nevertheless, PMRP showed obvious dose-effect relationship in the field of hair total melanin contents.

### 3.6. Effects of PMR and PMRP on Hair Growth Related Proteins and Factors

**Figure 7:**
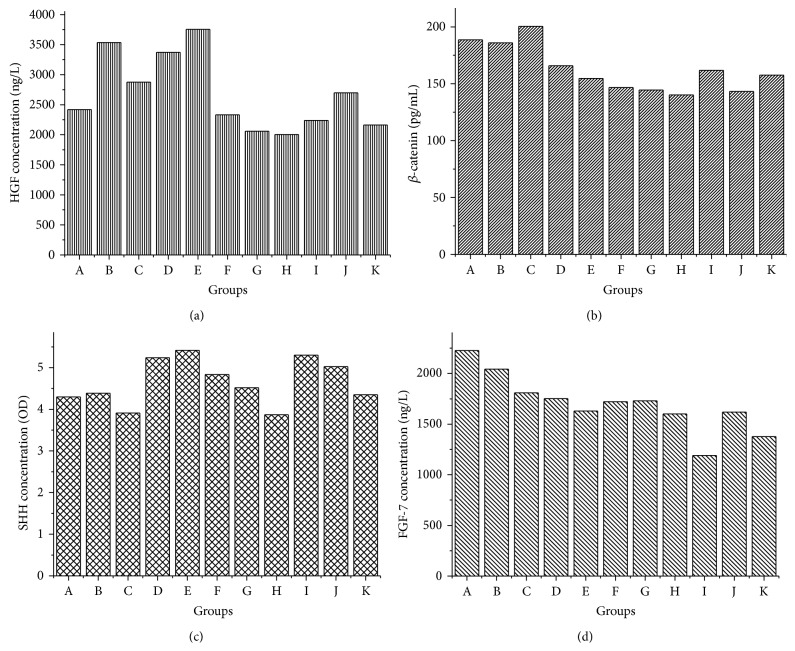
HGF (a), *β*-catenin (b), SHH (c), and FGF-7 (d) concentrations in the 6th week.

Concentrations of hair growth related proteins and factors in shaving skin could be correlated directly to the hair growth promotion activities. Therefore, major proteins and factors contents were tested in our research (shown in Figure 7).

As shown in Figure 7(a), treatment of PMR could increase expression of HGF in all dosage groups (Groups B to D). However, only low dosage PMRP accelerated the HGF expression. For topical dosage groups, HGF concentrations in both PMP and PMRP groups had no obvious change.

For the orally taken groups, *β*-catenin concentrations in three dosage groups of PMP and low dosage of PMRP were higher than the positive control group (Figure 7(b)). For external usage, *β*-catenin concentrations had no obvious change in all groups.

SHH contents in PMRP groups were higher than in the same dosage PMR groups (Figure 7(c)). Topical usage of PMRP (Group I) also gave out higher SHH content than topical usage of PMR (Group H). SHH gene reported to play an important role in accelerating hair follicles from telogen stage to anagen stage [7]. This indicated that the SHH expression was increased by PMRP.

In the 6th week, no obvious changes of FGF-7 contents were observed in all groups (Figure 7(d)), which indicated that the contents of FGF-7 were not directly related to the hair growth rate.

The expressions of these factors showed obvious time-effect relationship (see Supplementary Material, Tables S1 and S2 and Figure S1, available online at http://dx.doi.org/10.1155/2015/517901). HGF level consistently declined from 3rd to 6th week. The highest *β*-catenin, FGF-7, and SHH expressions were also found in the 3rd week. However, the highest IGF-1 level was observed in the 4th or 6th week, but not the 3rd week.

### 3.7. Relationships between Hair Growth Activity and Related Proteins and Growth Factors in Skin

Pearson's correlation coefficients between hair growth activity and related proteins and growth factors within groups were listed in Table 4. FGF-7 contents were highly positive correlated with hair covered skin ratio (*r* = 0.897, *p* < 0.05) in oral PMR groups. That indicated that the hair growth promotion effect of PMR was most probably mediated by the expression of FGF-7. This could be the key mechanism involved in the hair growth promotion activity of PMR.

## 4. Conclusions

**Figure 8:**
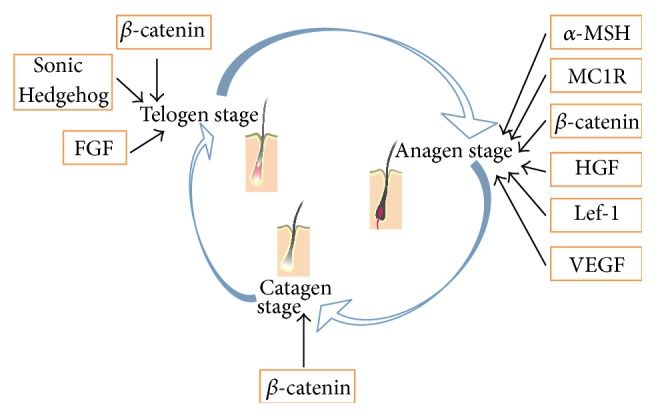
Major proteins and factors involved in the hair growth cycle.

Hair growth was genetically regulated by many proteins and factors [8] (Figure 8). *β*-catenin was an important component in Wnt signaling pathways, and it was indispensable to skin replacement and hair follicle development [9]. SHH could significantly facilitate telogen stage or catagen stage hair follicles entering into anagen stage, thus promoting hair growth [10]. HGF could facilitate hair follicles to enter the anagen stage when injected to newborn mice [11]. IGF-1, highly homologous with insulin, could promote hair follicle growth [12]. Li et al. [13] found that IGF-1 could maintain the hair follicles' growth in* in vitro* cultivation of human hair follicle cells. Many studies had found that FGF-7 shows obvious adjustment to the hair growth. FGF-7 could promote hair to enter the anagen stage and increase hair follicles amounts [11]. Vascular endothelial growth factor (VEGF), a multifunctional cytokine, could not only induce the formation of blood vessels around hair follicles, but also promote directly the dermal papilla cells proliferation [14]. Lymphatic enhancement factor (Lef-1) was a key nuclear transcription factor in the Wnt/*β*-catenin signaling pathways. A lot of studies had shown that whisker hair follicles disappear completely and back skin hair follicles growth stops in Lef-1 knockout mice. Therefore, Lef-1 played an important role in regulating the hair follicle growth [15]. Melanin receptor-1 receptor (MC1R) gene [16] was considered to be a crucial factor in human hair color and skin color. MC1R, expressed in the melanocyte or melanin cell tumor, was receptor of alpha-melanocyte stimulating hormone (*α*-MSH). After binding to MC1R, *α*-MSH could activate the adenylate cyclase (AC), which may lead to increasing intracellular cyclic adenosine monophosphate (cAMP). cAMP could activate tyrosinase enzyme though protein kinase A (PKA), a rate limiting enzyme. This was a classic way of synthesis of melanin by *α*-MSH in melanocyte [17].

In this research, system assessment of hair growth activity and possible mechanisms of PMR and its processed products PMRP were carried out. FGF-7, SHH, *β*-catenin, IGF-1, and HGF contents were measured from 3rd to 6th week.

Judging from our obtained results, we found both oral administration of PMR and topically given PMRP could promote hair growth. The hair growth promotion effect of oral PMR was most probably mediated by the expression of FGF-7, while topical PMRP promotes hair growth by the stimulation of SHH expression. This founding enhanced our confidence in natural products application in the field of hair promotion in the future.

A follow-up study with DHT overexpression animal model was needed for the complete illumination of the effects and mechanisms of PMR and PMRP in the treatment of AGA. Moreover, the active material basis was also required to be explained.

## Supplementary Material

Table S1: HGF and *β*-catenin concentration changes in all groups from 3rd to 6th week were listed in Table S1. Their expressions in the skin tissue may be in variation with the hair growth cycle. The highest expressions of HGF and *β*-catenin were observed in the 3rd week.
Table S2: FGF-7 and IGF-1 concentration changes in all groups from 3rd to 6th week were listed in Table S2. Their expressions in the skin tissue may be in variation with the hair growth cycle. The highest expressions of FGF-7 and IGF-1 were observed in the 3rd and 4th week, respectively.

## Figures and Tables

**Table 1:** Animal groupings and treatments.

Groups	Drug delivery route	Treatments	Dosage (g/kg)
A	Oral	Physiological saline	—
B	Oral	PMR	0.2925
C	Oral	PMR	0.5850
D	Oral	PMR	1.1700
E	Oral	PMRP	0.2925
F	Oral	PMRP	0.5850
G	Oral	PMRP	1.1700
H	Topical	PMR	0.5850
I	Topical	PMRP	0.5850
J	Oral and topical	PMR	0.5850
K	Oral and topical	PMRP	0.5850

**Table 2:** Hair length of mice in oral groups (*X* ± SD, mm, *n* = 10/per mice, *n* ≥ 3).

Groups	2nd week	3rd week	4th week	5th week	6th week
A	4.28 ± 1.14	9.22 ± 0.47	9.50 ± 0.50	10.24 ± 0.23	9.92 ± 0.47
B	5.09 ± 0.79	9.24 ± 0.78	9.78 ± 0.47	9.71 ± 0.05	10.05 ± 0.66
C	4.42 ± 1.03	9.81 ± 0.93	8.67 ± 0.65	9.84 ± 0.57	10.73 ± 0.49
D	3.96 ± 1.74	9.24 ± 0.60	10.06 ± 0.78	9.42 ± 0.65	10.76 ± 0.98
E	3.06 ± 0.47	8.69 ± 0.67	9.08 ± 0.89	10.05 ± 1.23	10.75 ± 1.39
F	3.66 ± 0.21	7.66 ± 0.92^*∗*^	8.93 ± 0.99	9.73 ± 0.63	10.35 ± 0.49
G	3.63 ± 1.77	7.94 ± 1.47	8.58 ± 0.60	7.99 ± 1.79	9.52 ± 0.70

The *∗* indicates a significant difference compared with control group. ^*∗*^ *p* < 0.05.

**Table 3:** Comparison between orally and topically administrated groups.

Treatments	Oral		Topical		Oral + topical	
	PMR	PMRP	PMR	PMRP	PMR	PMRP
Groups	C	F	H	I	J	K
Average hair length (mm)	10.73	10.35	10.44	10.24	9.86	9.35
Average hair covered skin ratio (%)	96.5%	66.82%	80.73%	89.51%	77.31%	91.59%
Total melanin (OD)	0.0875	0.0835	0.0945	0.0960	0.0950	0.0960
Follicles	13	7	8	10	12	9

**Table 4:** Person's correlation coefficients between hair growth activity and related factors.

Correlation coefficient		HGF	SHH	*β*-catenin	FGF-7	IGF-1
PMR oral groups	Hair length	—	—	—	—	—
	Total melanin	—	—	—	—	—
	Hair covered skin ratio	—	—	—	*r* = 0.897^*∗*^	—

PMRP oral groups	Hair length	—	—	—	—	—
	Total melanin	__	—	—	—	—
	Hair covered skin ratio	—	—	—	—	—

^*∗*^ *p* < 0.05.

## Abbreviations

AC: Adenylate cyclase
ATP: Adenosine triphosphate
cAMP: Cyclic adenosine monophosphate
DHT: Dihydrotestosterone
FGF-7: Fibroblast growth factor-7
HGF: Hepatocyte growth factor
HPLC-DAD: High performance liquid chromatography diode array detector
H&E: Hematoxylin and eosin
IGF: Insulin-like growth factor-1
Lef-1: Lymphatic enhancement factor-1
MC1R: Melanocortin-1 receptor
*α*-MSH: Alpha melanocyte stimulating hormone
PKA: Protein kinase A
PMR: * Polygonum multiflorum* Radix
PMRP: * Polygonum multiflorum* Radix Preparata
SHH: Sonic Hedgehog
TSG: 2,3,5,4′-Tetrahydroxystilbene-2-*O*-*β*-D-glucoside
VEGF: Vascular endothelial growth factor.
