# Supplementary material for: Hair Growth Promotion Activity and Its Mechanism of Polygonum multiflorum
Source: Evid Based Complement Alternat Med. 2015 Jul 30;2015:517901. doi: 10.1155/2015/517901 (PMC4534627; doi:10.1155/2015/517901)
Supplement: Supplementary file 1 — Table S1: HGF and β-catenin concentration changes in all groups from 3rd to 6th week were listed in Table S1. Their expressions in the skin tissue may be in variation with the hair growth cycle. The highest expressions of HGF and β-catenin were observed in the 3rd week. Table S2: FGF-7 and IGF-1 concentration changes in all groups from 3rd to 6th week were listed in Table S2. Their expressions in the skin tissue may be in variation with the hair growth cycle. The highest expressions of FGF-7 and IGF-1 were observed in the 3rd and 4th week, respectively. [file 517901.f1.pdf]

Table S1. HGF and β-catenin concentration changes from 3rd to 6th week (ng/l)

Groups	3rd week		4th week		6th week	
	HGF	β-catenin	HGF	β-catenin	HGF	β-catenin
A	4188.40	202.40	3252.00	209.70	2419.60	188.55
B	4506.75	190.75	3712.25	208.55	3535.05	186.05
C	4388.25	211.35	3577.45	230.80	2875.90	200.45
D	4774.45	181.15	4252.70	198.20	3372.15	165.80
E	4522.25	174.65	3683.15	169.00	3755.55	154.70
F	4412.70	100.90	3238.81	175.75	2332.00	146.60
G	4294.95	170.35	2705.10	175.45	2059.85	144.50
H	4216.40	165.80	3241.45	196.80	2006.20	140.15
I	4475.05	179.25	3118.45	164.95	2240.30	161.80
J	4118.60	198.20	4085.45	197.10	2699.35	143.25
K	3433.80	196.25	4142.75	161.30	2163.05	157.60

Table S2 FGF-7 and IGF-1 concentration changes from 3rd to 6th week (ng/l)

Groups	3rd week		4th week		6th week	
	FGF-7	IGF-1	FGF-7	IGF-1	FGF-7	IGF-1
A	2499.437	18626.97	2354.498	18747.84	2227.239	19634.11
B	2163.705	17233.48	2810.462	19473.22	2043.047	19458.06
C	1904.931	20665.12	2078.877	18977.08	1808.215	21353.46
D	2552.872	18611.15	1700.589	20088.97	1752.616	19090.72
E	2604.875	18243.99	1733.606	17886.78	1629.188	19208.82
F	2616.66	19563.89	1751.349	20010.69	1720.917	19946.83
G	2554.057	18857.58	1780.446	19848.13	1731.069	21024.58
H	2576.54	18992.61	2078.877	21056.86	1601.03	18945.97
I	2152.674	21199.03	1715.838	22157.90	1191.217	20360.26
J	2056.65	17870.40	2210.167	19341.48	1618.956	20432.21
K	2379.784	17766.40	2130.582	19639.11	1377.496	20079.21

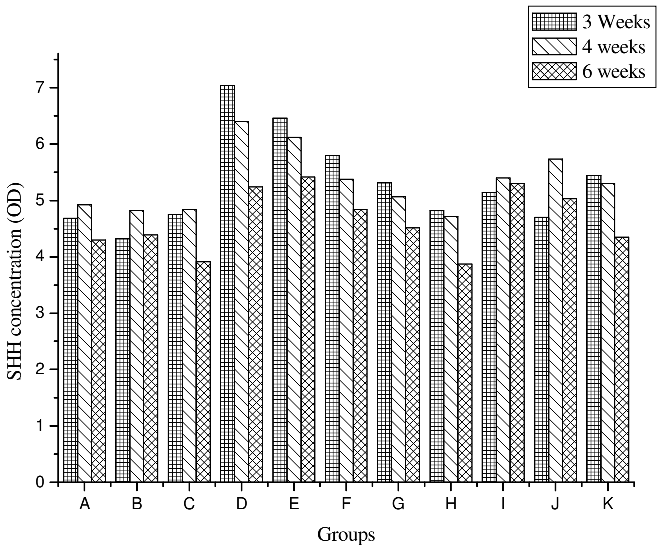

Figure S1. SHH concentration changes from 3rd to 6th week.
